# Cytokine pathway disruption in a mouse model of schizophrenia induced by Munc18-1a overexpression in the brain

**DOI:** 10.1186/1742-2094-11-128

**Published:** 2014-07-29

**Authors:** Itziar Gil-Pisa, Carolina Cebrián, Jorge E Ortega, J Javier Meana, David Sulzer

**Affiliations:** 1 Department of Neurology, Columbia University Medical Center, 710 W, 168th Street, New York, NY 10032, USA; 2 Department of Pharmacology, University of the Basque Country (UPV/EHU), Barrio Sarriena s/n, Leioa, Bizkaia 48940, Spain; 3 Centro de Investigación Biomédica en Red de Salud Mental (CIBERSAM), Barrio Sarriena s/n, Leioa, Bizkaia 48940, Spain; 4 BioCruces Health Research Institute, Plaza de Cruces, Barakaldo, Bizkaia 48903, Spain

**Keywords:** Munc18-1a, Animal model, Schizophrenia, Neuroinflammation, Cytokine

## Abstract

**Background:**

An accumulating body of evidence points to the significance of neuroinflammation and immunogenetics in schizophrenia, and an imbalance of cytokines in the central nervous system (CNS) has been suggested to be associated with the disorder. Munc18-overexpressing mice (Munc18-OE) have provided a model for the study of the alterations that may underlie the symptoms of subjects with schizophrenia. The aim of the present study was to elucidate the involvement of neuroinflammation and cytokine imbalance in this model.

**Methods:**

Cytokines were evaluated in the cortex and the striatum of Munc18-OE and wild-type (WT) mice by enzyme-linked immunosorbent assay (ELISA). Protein levels of specific microglia and macrophage, astrocytic and neuroinflammation markers were quantified by western blot in the cortex and the striatum of Munc18-OE and WT mice.

**Results:**

Each cytokine evaluated (Interferon-*gamma* (IFN-γ), Tumor Necrosis Factor-*alpha* (TNF-α), Interleukin-2 (IL-2) and CCL2 chemokine) was present at higher levels in the striatum of Munc18-OE mice than WT. Cortical TNF-α and IL-2 levels were significantly lower in Munc18-OE mice than WT mice. The microglia and macrophage marker CD11b was lower in the cortexes of Munc18-OE mice than WT, but no differences were observed in the striatum. Glial Fibrillary Acidic Protein (GFAP) and Nuclear Factor-*kappa*B (NF-κB)p65 levels were not different between the groups. Interleukin-1*beta* (IL-1β) and IL-6 levels were beneath detection limits.

**Conclusions:**

The disrupted levels of cytokines detected in the brain of Munc18-OE mice was found to be similar to clinical reports and endorses study of this type for analysis of this aspect of the disorder. The lower CD11b expression in the cortex but not in the striatum of the Munc18-OE mice may reflect differences in physiological activity. The cytokine expression pattern observed in Munc18-OE mice is similar to a previously published model of schizophrenia caused by maternal immune activation. Together, these data suggest a possible role for an immune imbalance in this disorder.

## Background

Inflammation is a complex response of the host to tissue injury such as an infection or a physical insult [1]. Cytokines are a family of proteins that mediate host response to infection, and are considered to be markers of infectious and inflammatory conditions [2,3]. Cytokines further mediate the cross-talk between the central nervous system (CNS) and the immune system, with consequences relevant to clinical psychiatry [4]. Over a decade ago, the CNS was formerly regarded to be effectively isolated from the peripheral immune system by the blood-brain barrier (BBB), but presently there is no doubt that cytokines from the peripheral immune system can invade the CNS under particular physiological and pathological conditions [5], and that multiple cytokines are synthesized by resident cells of the CNS including microglia, astrocytes and neurons [6-11].

Schizophrenia, a chronic and debilitating illness that affects about 1% of the world population [12], has recently been associated with increased levels of cytokines in the CNS [13-17]. An animal model of schizophrenia that features such aberrant cytokine handling would provide a tool for elucidating roles in the pathogenesis of this disease and exploring novel avenues of treatment based on immunomodulation.

Neurotransmitter release occurs mainly by exocytosis of secretory vesicles. It has been previously shown that cytosolic Sec/Munc (SM) proteins are involved in the assembly of the soluble NSF-attachment protein receptor (SNARE) complex and in the membranes attachment [18]. We have elected to study abnormalities of the Munc18-1 SM protein that have been implicated in the pathogenesis of schizophrenia [19,20]. Munc18-1 participates in SNARE interactions and plays an important role in the steps leading to synaptic vesicle exocytosis [21,22]. *Postmortem* proteomic analysis of the prefrontal cortex of subjects with schizophrenia demonstrates abnormally high Munc18-1 protein levels in membrane microdomains of the gray matter [23]. In addition, antipsychotic drug treatment seems to be associated with a lower content of key proteins of the exocytotic machinery, which could result in the destabilization and/or impairment of neuroexocytosis [24]. Alternative splicing of the gene coding for Munc18-1 results in two variants, the long isoform Munc18-1a (brain and retina) and the ubiquitous short isoform Munc18-1b [25,26]. Based on these premises, a transgenic mouse overexpressing the brain-specific isoform protein Munc18-1a (Munc18-OE mouse) was developed to assess whether abnormalities in exocytosis machinery could lead to a schizophrenic phenotype [27]. Urigüen *et al*. demonstrated that the overexpression of Munc18-1a produces a spectrum of alterations that resemble schizophrenic symptoms and/or neurobiological findings observed in subjects with schizophrenia [27], including a deficit in prepulse inhibition (PPI) and social functioning [28,29]. Moreover, Munc18-OE animals displayed dopaminergic dysfunction [27], which has been proposed as a crucial component of the pathogenesis of schizophrenia [30].

The objective of the present study is to determine if there are signs of neuroinflammation in the Munc18-OE mouse model. A range of cytokines previously described to be disrupted in the blood or cerebrospinal fluid (CSF) of patients with schizophrenia [14-17] were measured in the cortex and the striatum of Munc18-OE mice and corresponding WT controls. We further determined levels of specific microglia and macrophage and astrocytic markers and NF-κB (p65), a multifunctional transcription factor that is an important mediator of inflammation [31].

## Methods

### Animals

Assays were performed on adult (between 8 and 16-weeks-old) male C57BL6/CBA mice (National Institute for Agronomic Research, Madrid, Spain). Munc18-OE mice were generated as previously described by pronuclear microinjection [27]. WT mice (National Institute for Agronomic Research, Madrid, Spain) used as controls displayed the same background. A total of n = 5 animals per group and experiment were used. All animals were housed under a controlled temperature (22 ± 1°C), at a 12 hour light/dark cycle (lights on: 7:30 a.m. to 7:30 p.m.), and humidity (between 65 and 70%) with food and water *ad libitum.* Mice were killed by cervical dislocation and their brains were quickly frozen at −80°C. The day of the experiment, the whole cerebral cortex and the dorsal striatum of each mouse were dissected at 4°C. All animal procedures were performed in accordance with the European Directive for the Protection of Vertebrate Animals used for Experimental and Other Scientific Purposes (European Union Directive #86/606/EEC) and approved by the UPV/EHU Ethical Board for Animal Welfare (CEBA) and Genetically Modified Organisms (CEIAB). More recently, new regulations for research procedures with animals have been established (Royal Decree 53/2013).

### Enzyme-linked immunosorbent assay (ELISA)

Samples were homogenized on ice using a Sonic Dismembrator 60 (Thermo Fisher Scientific Inc., Massachusetts, United States) in a cytokine extraction buffer containing 0.05% bovine serum albumin (BSA), 0.02 mg/ml aprotinin, 0.02 mg/ml phenylmethanesulfonyl fluoride (PMSF), 0.05 mg/ml benzethonium chloride, 0.5% polyoxyethylene 20 sorbitan monooleate or Tween 20 (purchased from Sigma Aldrich^©^, Missouri, United States), 0.37 mg/ml ethylenediaminetetraacetic acid (EDTA) and 0.023 mg/ml sodium chloride (NaCl) (purchased from Merck, Darmstadt, Germany) followed by centrifugation at 10,000 rpm for 10 minutes at 4°C. The cytokine levels were assessed in the supernatants by ELISA, according to the manufacturer’s instructions (eBioscience Inc., California, United States). All samples were assayed at a dilution 1:2 in the buffer and the assays sensitivities were respectively: 0.7 to 100 pg/ml IFN-γ, 8 to 1,000 pg/ml TNF-α, 8 to 1,000 pg/ml IL-1β, 2 to 200 pg/ml IL-2, 4 to 500 pg/ml IL-6 and 15 to 2,000 pg/ml CCL2 chemokine. The Beckman Coulter DTX 880 Multimode detector (Beckman Coulter Inc., Califronia, United States) was used to determine the concentration of cytokines. Sample cytokine levels were determined using standard concentration curves evaluated in duplicate for each plate. Data were normalized to sample protein concentration, as determined by the Pierce BCA Protein Assay Kit (Thermo Scientific Inc., Massachusetts, United States). Values were expressed in pg cytokine/mg protein.

### Immunoblot assays

Samples were homogenized on ice using a Sonic Dismembrator 60 in a lysis buffer containing 50 mM Tris-HCl buffer (pH = 7.4) (Sigma Aldrich^©^, Missouri, United States), 150 mM NaCl, 5 mM EDTA and 1% Triton (Sigma Aldrich^©^, Missouri, United States). The protein concentration was determined with Pierce BCA Protein Assay Kit, using BSA as a standard. Samples were diluted in a loading buffer containing 70 mM Tris-HCl (pH = 6.8), 2% sodium dodecyl sulfate (SDS), 120 mM dithiothreitol, 6% glycerol and 0.0025% bromophenol blue (purchased from Sigma Aldrich^©^, Missouri, United States). Samples were denatured at 95°C for 5 minutes and 20 μg of total protein were loaded on each well. Proteins were separated by electrophoresis in SDS polyacrylamide gels and transferred to polyvinylidene fluoride membranes. Membranes were blocked for 1 hour at room temperature with Tris-HCl**-**buffered saline solution containing 5% nonfat dried milk (Sigma Aldrich^©^, Missouri, United States) and 0.05% Tween 20. Membranes were then incubated at 4°C overnight with specific primary antibodies: rabbit monoclonal antibody anti-CD11b (NB110-89474, Novus Biologicals, Colorado United States) at a dilution of 1:1,000, rabbit polyclonal antibody anti-glial fibrillary acidic protein (GFAP) (ab7260-50, lot No. 740637, Abcam®, Cambridge, England) at a dilution of 1:50,000, and rabbit polyclonal antibody anti-NF-κB (NB100-78423, lot No. B128550, Novus Biologicals, Colorado, United States) at a dilution of 1:500. As a control for sample loading and protein transfer, the blots were stripped and re-probed with monoclonal antibody anti-β-actin (A5441, lot No. 061 M4808, Sigma Aldrich^©^, Missouri, United States) at a dilution of 1:20,000. The membranes were incubated with appropriate horseradish peroxidase (HRP)-conjugated secondary antibodies (Thermo Scientific, Massachusetts, Unites States) at a dilution of 1:20,000 in blocking solution for 1 hour at room temperature and developed with the chemiluminescence kit Immobilon™ Western HRP Sustrate (Millipore, Massachusetts, United States). Target proteins were quantified by densitometric scanning of specific immunoreactive bands (integrated optical density (IOD) units). The immunoreactivity value of the target proteins in each sample was first corrected with the corresponding value of β-actin and then calculated as a percentage of the corresponding mean value of the WT group, which was used as a reference value (100%).

### Data analysis

Data were analyzed with GraphPad Prism™ version 5.0 (GraphPad Software, California, United States) and InVivoStat (InVivoStat Statistical Software, United Kingdom). Results were expressed as mean ± SEM. Data were analyzed by Student’s *t*-test. All tests were two-tailed. The level of significance was set to *P* <0.05.

## Results

### Brain cytokine levels

**Figure 1:**
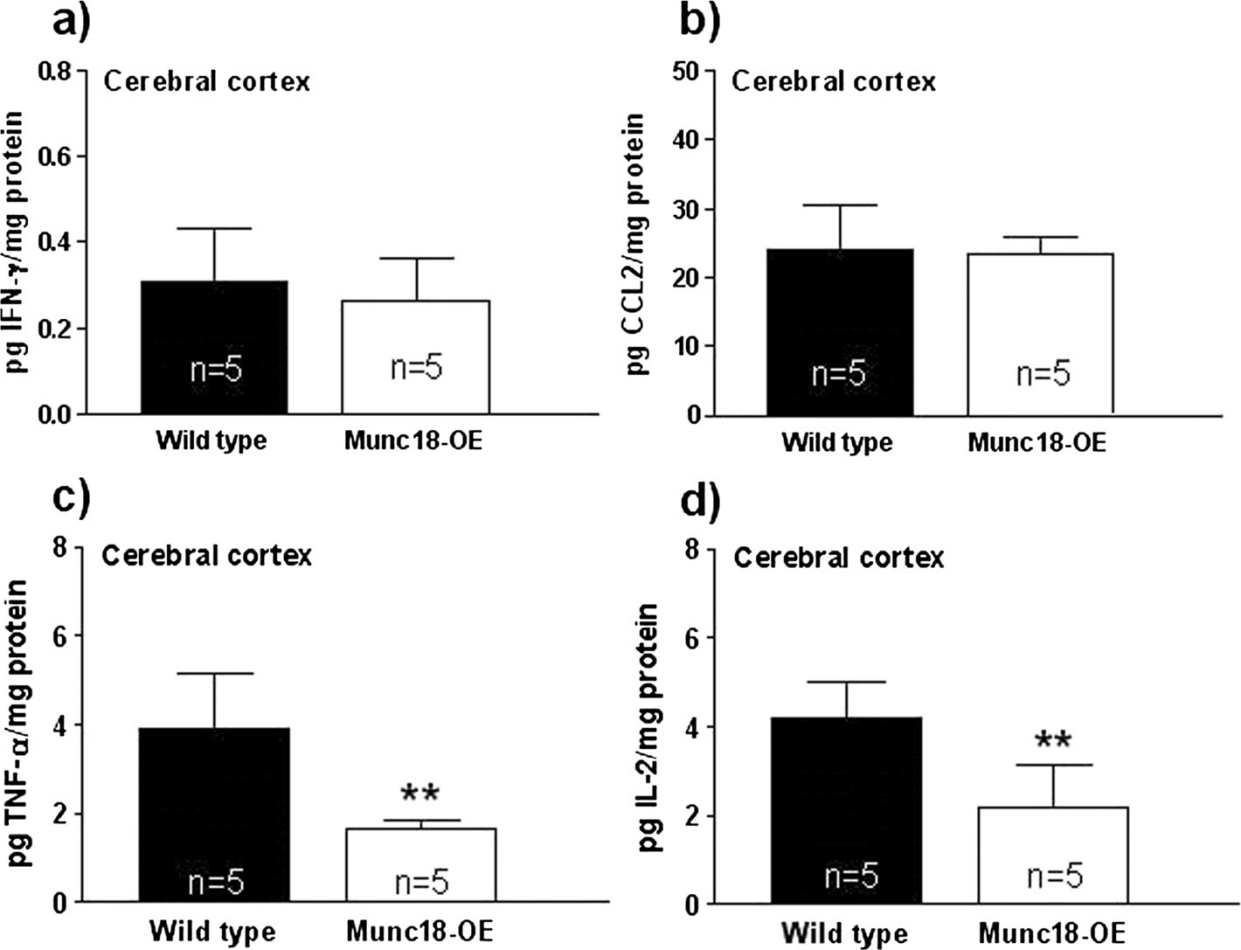
**Concentration of (a) IFN-γ, (b) CCL2, (c) TNF-α and (d) IL-2 in cortical homogenate from Munc18-OE (n = 5) and wild-type (n = 5) mice.** IFN-γ and CCL2 cortical levels were not different between groups. However, Munc18-OE mice showed a significant decrease in TNF-α and IL-2 cortical concentrations (*t* = 4.03, *P* <0.01; *t* = 3.62, *P* <0.01, respectively) compared to the WT. Data are expressed in pg cytokine/mg protein.

In the brain cortex samples there were no differences between groups in the levels of IFN-γ (Figure 1a) or CCL2 (Figure 1b). However, cortical TNF-α (Figure 1c) and IL-2 levels (Figure 1d) were significantly lower in Munc18-OE mice than WT (*t* = 4.03, *P* <0.01; *t* = 3.62, *P* <0.01, respectively).

**Figure 2:**
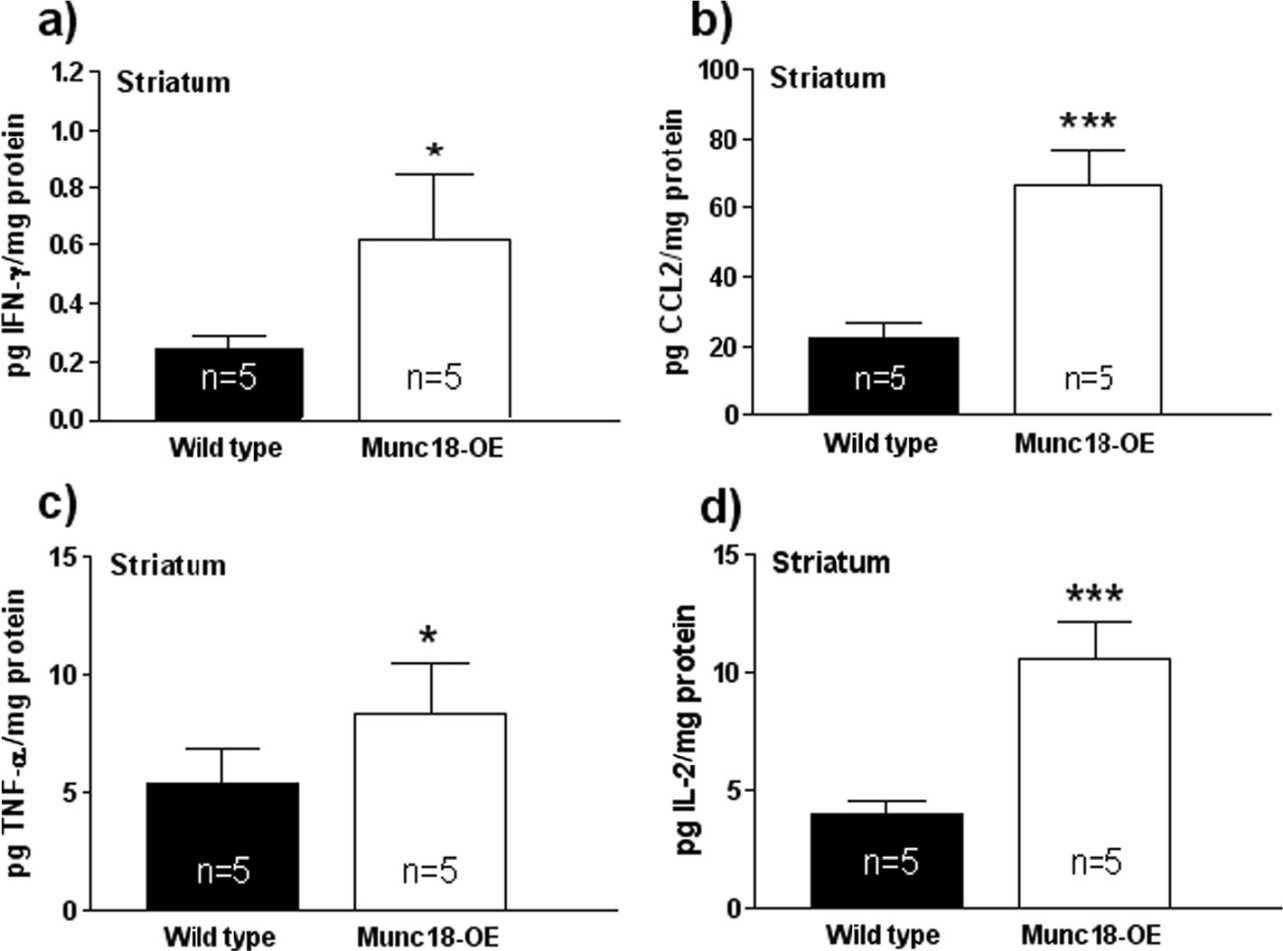
**Concentration of (a) IFN-γ, (b) CCL2, (c) TNF-α and (d) IL-2 in striatal homogenate from Munc18-OE (n = 5) and wild-type (n = 5) mice.** All cytokines were significantly increased in the striatum of Munc18-OE mice compared to WT mice showing significant differences between groups: IFN-γ (*t* = 2.91, *P* <0.05), CCL2 (*t* = 8.24, *P* <0.0001), TNF-α (*t* = 2.52, *P* <0.05), IL-2 (*t* = 8.51, *P* <0.0001). Data are expressed in pg cytokine/mg protein.

Interestingly, all the evaluated cytokines were significantly increased in the striatum of Munc18-OE mice compared to WT mice (Figure 2): IFN-γ (*t* = 2.91, *P* <0.05), CCL2 (*t* = 8.24, *P* <0.0001), TNF-α (*t* = 2.52, *P* <0.05), IL-2 (*t* = 8.51, *P* <0.0001).

The levels of IL-1β and IL-6 were under detection limits (8 pg/ml and 4 pg/ml, respectively) in both the cortex and striatum of Munc18-OE and WT mice (data not shown).

### Brain inflammatory markers

The microglia and macrophage marker CD11b was identified by western blot as a single band of 160 kDa, GFAP as a band of 55 kDa and the NF-κB p65 subunit as a band of 65 kDa.

**Figure 3:**
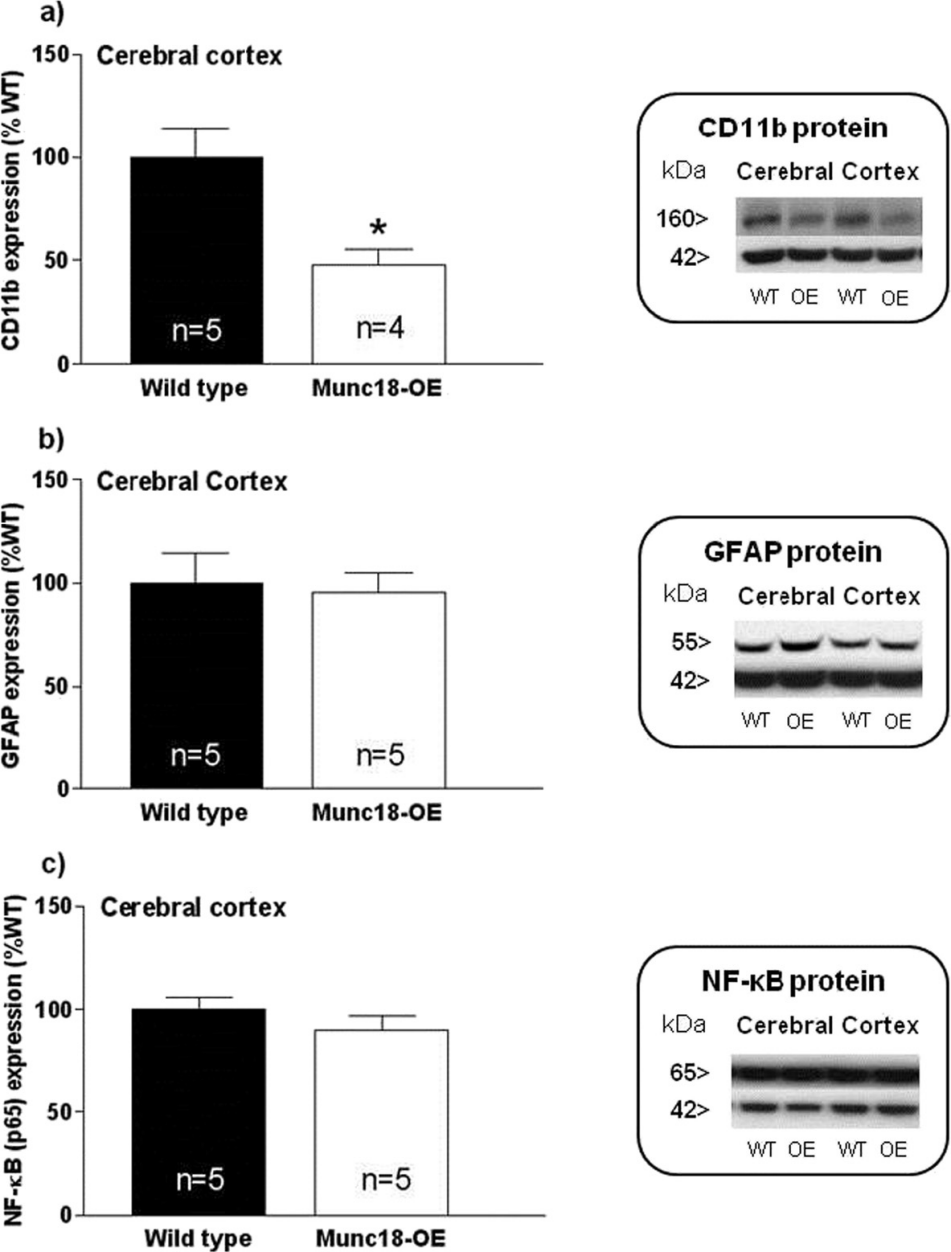
**Immunodensities of (a) CD11b, (b) GFAP and (c) NF-κB (p65) proteins with representative immunoblots in cerebral cortex from Munc18-OE (n = 5) and wild-type (n = 5) mice.** Bar graphs are ratios of optical densities of our proteins of interest to β-actin (42 kDa band), expressed as immunoreactivity in percentage of the mean value of the WT group (100%). CD11b was significantly decreased in Munc18-OE mice compared to the WT (*t* = 3.01; *P* <0.05). No differences between groups were observed in the levels of GFAP or NF-κB. Right panels are representative immunoblots for target proteins and β-actin which included Munc18-OE (OE) and wild-type (WT) mice samples. The molecular masses were estimated from referenced standards.

**Figure 4:**
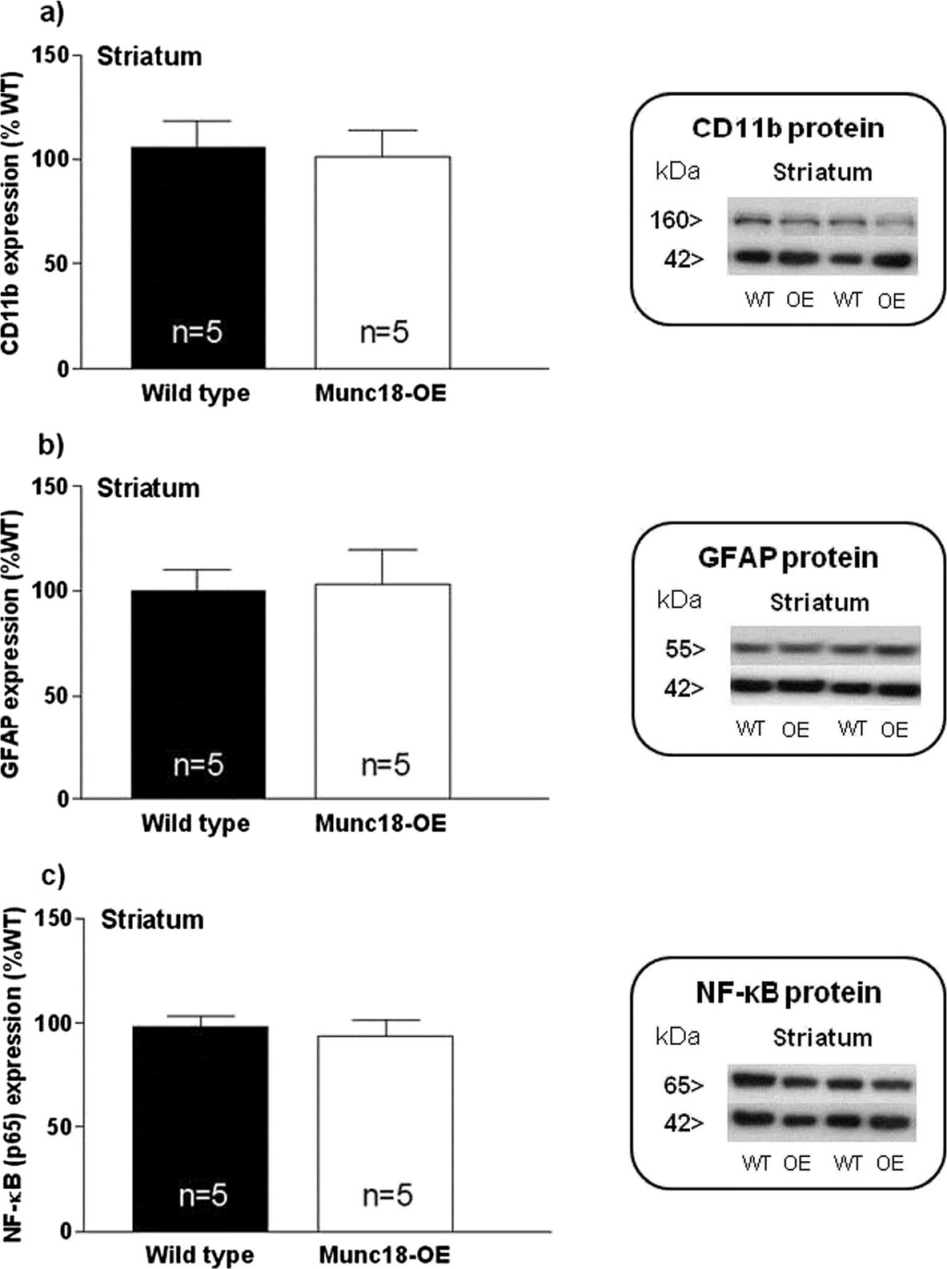
**Immunodensities of (a) CD11b, (b) GFAP and (c) NF-κB (p65) proteins with representative immunoblots in striatum from Munc18-OE (n = 5) and wild-type (n = 5) mice.** Bar graphs are ratios of optical densities of our proteins of interest to β-actin (42 kDa band), expressed as immunoreactivity in percentage of mean value of the WT group (100%). No differences were detected between groups for any of the analyzed proteins. Right panels are representative immunoblots for target proteins and β-actin which included Munc18-OE (OE) and wild-type (WT) mice samples. The molecular masses were estimated from referenced standards.

In the cortical samples, we observed lower CD11b expression (Figure 3a) in Munc18-OE mice than WT mice (*t* = 3.01, *P* <0.05), whereas no differences were detected in the levels of GFAP between both groups (Figure 3b). Although no statistically significant changes were obtained in the NF-κB transcription factor p65 subunit between both groups of mice, a trend of lower levels in Munc18-OE mice was observed (Figure 3c).None of these inflammatory parameters protein levels differed in the striatum of Munc18-OE from those quantified in WT mice (Figure 4).

## Discussion

Schizophrenia is a syndrome with multiple etiologies and symptoms, and development of relevant animal models has relied on focusing on specific features associated with schizophrenia, rather than mimicking the entire syndrome. As previously described [27], the overexpression of Munc18-1a in mice resulted in a successful replication of several behavioral, neurochemical and morphological features associated with schizophrenia. Here we show that in addition to this, the Munc18-OE mice exhibit a neuroinflammatory pattern that is similar to the one reported in the genuine disease.

Interestingly, we found that IL-2 and TNF-α cortical levels are lower in transgenic compared to WT mice, and that the levels of microglia and macrophage marker CD11b are also decreased in the cortex of Munc18-OE mice. The decreased TNF-α in the cortex of Munc18-OE mice could be responsible for reduced microglia activation displayed by decreased microglia and macrophage marker CD11b expression. Furthermore, IL-2 cytokine, which was also decreased in the cortex, is mostly synthesized by activated microglia in the CNS [32]. In contrast, a non-statistically significant trend to reduced NF-κB was observed, mainly in the cortex. Taking into account the sample size, a type II error cannot be discarded for the differences between groups obtained in the cortex and the striatum for NF-κB. Thus, the non-significant decrease displayed by the Munc18-OE animal model might be relevant to schizophrenia, a disease associated with increased NF-κB levels in peripheral tissues of schizophrenic subjects. However, these alterations in the cytokine network in schizophrenia might be secondary to antipsychotic treatment, which has an important impact on the immune system [33,34]. Indeed, the status of NF-κB in drug-free schizophrenic subjects remains to be elucidated. Globally, the present results could be suggesting a decrease in proinflammatory pathways in the cerebral cortex of Munc18-OE mice compared to WT mice.

It is remarkable that the levels of proinflammatory cytokines are decreased in the cortex of transgenic mice compared to WT, whereas these levels are increased in the striatum. This would be consistent with previous data that indicate opposite or compensatory effects to the cortex and striatum of schizophrenic patients [35,36].

A growing body of evidence shows that many aspects of the symptoms of schizophrenia could be ascribed to the disruption of the prefrontal cortex (hypofrontality) [37-39]. This phenomenon could involve not only hypofunctional areas but areas of overactivity as well, such as temporal or limbic areas [37]. Furthermore, it has been suggested that the cortical state underlies the negative symptoms and cognitive impairments in schizophrenia, contributing to the typical poor social outcome of this disease, while the subcortical state underlies the positive symptoms. Additionally, there are findings consistent with the notion that impaired prefrontal dopamine (DA) transmission may be related to hypofrontality [40]. Furthermore, this hypodopaminergic state in the cortex may produce a subsequent hyperdopaminergic state in the subcortical regions [35,41,42], due to the fact that cortical pathways exert an inhibitory control of subcortical DA transmission [43,44]. Functional imaging studies in schizophrenic patients have shown reduced frontal lobe activity, that is, reduced glucose utilization and decreased regional cerebral blood flow [38,45-47]. Decreased levels of proinflammatory cytokines in the cortex, followed by an increase of these cytokines in the striatum, could be a compensatory mechanism similar to the compensatory hyperactivity observed in schizophrenic patients.

Additionally, immune and inflammatory processes in prenatal and perinatal stages are suggested to play crucial roles in the vulnerability to schizophrenia. Munc18-OE mice show symptoms similar to those induced in adult offspring by the polyinosinic:polycytidylic acid (poly I:C) maternal immune activation model of schizophrenia [48-50]. It would be interesting to evaluate neuroinflammation in other animal models to elucidate whether this potential correlation between a disruption in the levels of proinflammatory markers and schizophrenia is present.

As above, CNS measured cytokines may originate native or infiltrated cells in the CNS or peripheral cytokines could be transported across the BBB [51,52]. While we find that the brain of Munc18-OE mice presents an imbalance of immune and inflammatory systems, further studies including activity experiments should be carried out to identify the sources of the altered inflammatory signals observed in transgenic mice.

In summary, the overexpression of Munc18-1a results in an imbalance of immune and inflammatory systems, which may be induced directly or indirectly. Multiple secretory pathways for cytokines have been characterized, including regulated exocytosis which involves SNARE and SM proteins [53]. Furthermore, Munc18-OE mice displayed dopaminergic dysfunction, consistent with reports that neurotransmitters may modulate microglia-mediated neuroinflammation [54]. Nevertheless, further studies are necessary to elucidate the mechanism leading from Munc18-1a overexpression to the disrupted immune and inflammatory systems.

We note that immunological and inflammatory parameters related to schizophrenia have been determined mainly in peripheral human samples such as blood and CSF [14-17], and there is a dearth of data in levels of proinflammatory markers in human brain tissue, which will be required to provide insight into the relevance of these models.

## Conclusions

The cytokine levels measured in the brains of Munc18-OE mice are consistent with data obtained in patients with schizophrenia that indicate hypofrontality and striatal overactivity. The Munc18-OE mice displayed similar symptoms to those induced by maternal immune activation, suggesting a potential convergence of immune imbalance in the schizophrenic phenotype derived from multiple etiologies. It is noteworthy that these findings are consistent with the growing evidence that immunity and inflammatory imbalance seems to be related to the pathophysiology of schizophrenia [55].

## Abbreviations

BBB: Brain blood barrier; BSA: Bovine serum albumin; CSF: Cerebrospinal fluid; CNS: Central nervous system; DA: Dopamine; ELISA: Enzyme linked immunosorbent assay; GFAP: Glial fibrillary acidic protein; IFN: Interferon; IL: Interleukin; Munc18-OE: Munc18-overexpression; NF-κB: Nuclear factor-kappaB; SDS: Sodium dodecyl sulfate; SM: Sec/Munc; SNARE: Soluble NSF-attachment protein receptor; TNF: Tumor necrosis factor; WT: Wild-type.

## Competing interests

JJM is co-author of a patent (Registration number: WO/2010/020642) related to Munc18-1 in schizophrenia owned by Brainco Biopharma SL and UPV/EHU. All other authors declare that they have no competing interests.

## Authors’ contributions

IGP and CC carried out the western blot and ELISA studies and performed the statistical analysis. CC and DS participated in the research project design. JEO and JJM have made substantial contributions to interpretation of data. IGP wrote the manuscript. CC, JEO, JJM and DS have been involved in drafting the manuscript and revising it critically for important intellectual content. All authors read and approved the final manuscript.
